# Cardiovascular health based on the life's essential 8: overall status and subgroup differences across sociodemographic characteristics in the Netherlands

**DOI:** 10.1016/j.pmedr.2026.103674

**Published:** 2026-10-23

**Authors:** Lieke M. Steendam, Ilonca Vaartjes, Raymond H. Wimmers, Yvonne Koop, Dutch Heart Foundation Cardiovascular Health Working Group (Appendix B)

**Affiliations:** a Julius Center for Health Sciences and Primary Care, University Medical Center Utrecht, Utrecht University, Utrecht, the Netherlands; b Dutch Heart Foundation, The Hague, the Netherlands

**Keywords:** Cardiovascular health, Life's essential 8, Risk factors, Diversity, Sociodemographic characteristics, Prevention, Real world data

## Abstract

**Objective:**

Estimating the cardiovascular health (CVH) status based on the Life's Essential 8 (LE8) in the Netherlands and determine subgroup differences across sociodemographic characteristics.

**Methods:**

Data from 2022 were obtained from two sources. Data on the behavioural factors diet, physical activity, nicotine exposure, sleep, and the health factor BMI were obtained from Statistics Netherlands. Data on the remaining health factors blood pressure, lipids, and glucose were obtained from a national primary care register. Total and metric specific LE8 mean scores were calculated, standardised, and stratified by sex, and, if the data allowed, by age, education, income, and migration background.

**Results:**

The mean LE8 score was 73.0/100, with 69.4 for behaviour factors and 76.5 for health factors. Individual metric scores ranged from 42.8 (diet) to 90.5 (physical activity). Women scored higher than men on all metrics except sleep and physical activity. Individuals with lower incomes or primary education had lower scores than individuals with higher incomes or tertiary education. No consistent pattern was observed across metrics by age and migration background, although characteristics in metric scores did vary.

**Conclusions:**

The CVH in the Netherlands is intermediate. Heterogeneity in the scores indicates the need for stratification and provides information to tailor interventions.

## Highlights

• The overall cardiovascular health is intermediate in the Netherlands.
• Individuals with lower incomes tend to have lower cardiovascular health scores.
• Individuals with primary education tend to have lower cardiovascular health scores.
• Heterogeneity in cardiovascular health scores for migration background and age.
• Heterogeneity should be reflected in research, healthcare, and interventions.

## 1 Introduction

Cardiovascular disease (CVD) is a major global public health burden, with an estimated 19.8 million people deaths in 2022 (32% of all deaths worldwide) (World Health Organization WHO, n.d.). This number is expected to rise between 2025 and 2050 due to the aging population (Chong et al., 2025). To reduce the CVD burden, increasing emphasis is placed not only on addressing traditional risk factors but also on lifestyle improvements (Eckel et al., 2014). Reflecting this shift of promoting health and prevention alongside addressing disease constitutes the concept of ideal cardiovascular health (CVH), introduced by the American Heart Association (AHA) in 2010 (Lloyd-Jones et al., 2010).

To measure ideal CVH, the AHA developed Life's Simple 7: seven factors for preventive health improvement, including dietary quality, physical activity, nicotine exposure, body mass index (BMI), cholesterol level, glucose level, and blood pressure (Lloyd-Jones et al., 2010). This combination of health and behaviour factors was carefully selected by a committee, based on extensive research identifying well-established risk factors for CVD (Lloyd-Jones et al., 2010). In 2022, the AHA added an eighth factor, sleep health, because both sleep quantity and quality are associated with CVD risk (Lloyd-Jones et al., 2022a). Together, the eight factors are referred to as Life's Essential 8 (LE8) (Lloyd-Jones et al., 2022a). The LE8 can serve as a framework to score CVH; where a score below 50 reflects a poor CVH, 50–79 an intermediate CVH, and 80 or above an ideal CVH (Lloyd-Jones et al., 2022a).

Previous studies consistently showed that higher or ideal CVH are associated with lower risk of cardiovascular events, increased CVD-free years, and increased life expectancy (Ma et al., 2023; Sebastian et al., 2025; Song et al., 2024). While the benefits are evident, LE8 scores vary across sociodemographic characteristics, including sex, ethnicity, and socioeconomic position. Women generally score higher on health factors, while men score higher on behaviour factors (Kaur et al., 2024). Younger adults tend to have higher LE8 scores compared to older adults (Deraz et al., 2025). Higher income and education level are associated with higher LE8 scores (Williams et al., 2023). In immigrant groups, CVH varies; a United States (US) population reported that Asian adults had the highest LE8 scores, followed by White, Latino/Hispanic, and African American individuals, with little change over time (Yang et al., 2025). The variation across sociodemographic characteristics highlights the need to consider heterogeneity when reporting CVH.

Up to now, information on LE8 based on representative data is lacking for the Netherlands. Obtaining this information may help to monitor CVH in the country and serve as a benchmark for other high-income countries to compare population-level CVH. Besides, stratifying by sociodemographic characteristics may provide information for tailored interventions, with the goal of reducing the burden of CVD. Therefore, this study aims to determine the nationwide (adults 18+) and subgroup CVH in the Netherlands using the LE8 framework.

## 2 Methods

### 2.1 Study design and population

We conducted a descriptive study with a cross-sectional design to estimate the LE8 CVH in the Netherlands in 2022. All individuals aged 18 years or older who were registered with one of the participating primary health care practices in 2022 and/or completed the 2022 Statistics Netherlands Health Questionnaire were included in the study.

### 2.2 Data sources

To estimate the CVH, we employed two national data sources: Netherlands Institute for Health Services Research (Nivel, in Dutch) and Statistics Netherlands (CBS, in Dutch).

#### 2.2.1 Nivel: blood lipids, blood glucose, and blood pressure

Health data were obtained from the Nivel Primary Care Database (Nivel-PCD), a database with routinely recorded data extracted from electronic health record (EHR), on primary health care practices in the Netherlands (*n* = 430 in 2022, covering 1,902,017 patients) (Heins et al., n.d.). The age and sex distribution of the patients closely matches national demographics. Data on laboratory measurements of blood lipids level (non-HDL cholesterol), blood glucose level (fasting blood glucose (FBG) for diabetics or HbA1c for non-diabetics), and blood pressure status were provided by Nivel. When multiple measurements were available, the most recent measurement was selected. The International Classification of Primary Care (ICPC)-1 was used for diagnosis (K85-K87, T90, T93). The data were aggregated and presented by biological sex and age groups (18–19, 85+, and the ages between 20 and 84 were categorised into 10-year strata).

#### 2.2.2 CBS: BMI, diet, nicotine exposure, physical activity, and sleep

Behaviour data and BMI were obtained from CBS. CBS collects detailed data on numerous societal and health-related themes, using data from surveys, administrative records, and nationwide registries. Data were obtained from the CBS Health Questionnaire, which is annually administered, using a stratified random sampling design, to a representative sample of the Dutch population (*n* = 8027 in 2022) (Centraal Bureau voor de Statistiek (CBS), 2014). We linked these data with the CBS national Population Register using a unique identifier. The Population Register is based on municipal records with demographic information for every registered resident of the Netherlands (Prins, 2017). Linkage to the Population Register allowed us to obtain information about education, income, and migration background.

### 2.3 Measures

#### 2.3.1 Health and behaviour factors

The LE8 metrics can be divided into health factors (BMI, blood lipids, blood glucose, and blood pressure) and behaviour factors (physical activity, diet, sleep, and nicotine exposure). To quantify each factor, we employed the framework developed by the AHA (Lloyd-Jones et al., 2022a). Some adjustments were made in the quantification to match the available Dutch data. These adjustments were based on literature and with consensus of the multidisciplinary Dutch Heart Foundation Cardiovascular Health Working Group (Appendix B). Detailed quantification, measurement, and scoring methods are provided in Table 1.

**Table 1:** Overview of the Life's Essential 8 metric quantification used in this study for determining the Cardiovascular Health status of the Dutch adult (18+) population, including the data source, method of measurement and stratification.

Domain	Life's Essential 8 Metric	Data source	Method of measurement	Quantification/scoring of LE8 metric	Stratification				
					Biological sex	Age category	Household income	Education level	Migration background
**Behaviour factors**	Diet	CBS Health questionnaire	Self-reported daily intake of fruits, vegetables, fish and alcohol.	Categories: Fruits ≥200 g/day Vegetables ≥250 g/day Fish ≥ once a week Alcohol ≤1/day **Table:** Points Number of categories met 0 0 20 1 50 2 80 3 100 4 Individuals with one or more missing categories, were excluded.	x	x	x	x	x
	Physical Activity	CBS Health questionnaire	Self-reported minutes of moderate or vigorous physical activity per week (SQUASH).	**Table:** Points Minutes/week 0 0 20 1–29 40 30–59 60 60–89 80 90–119 90 120–149 100 ≥ 150	x	x	x	x	x
	Nicotine exposure	CBS Health questionnaire	Self-reported use or previous use of cigarettes or inhaled nicotine-delivery system (NDS), and second-hand smoke exposure.	**Table:** Points Level 0 Current smoker 25 Former smoker, quit <1 years ago 50 Former smoker, quit 1 ≤ 5 years ago 75 Former smoker, quit ≥5 years ago or currently using inhaled NDS 100 Never smoker For individuals currently living with an active indoor smoker, 20 points were subtracted (unless the score was 0).	x	x	x	x	x
	Sleep health	CBS Health questionnaire	Self-reported problems with sleep	**Table:** Points Sleeping problems 0 Quite a lot 20 A lot 50 Quite 80 A bit 100 Non *‘To what extent have you suffered from sleeping problems in the past 2 weeks? Think of difficulty falling asleep, difficulty staying asleep, or waking up too early*.’	x	x	x	x	x
**Health factors**	BMI	CBS Health questionnaire	Self-reported body weight and height	**Table:** Points BMI (kg/m^2^) 0 ≥40.0 15 35.0–39.9 30 30.0–34.9 70 <18.5 or 25.0–29.9 100 ≥18.5 - <25	x	x	x	x	x
	Blood lipids	Nivel-PCD	Non-HDL cholesterol from a blood sample.	**Table:** Points Non-HDL-cholesterol (mmol/L): 0 ≥5.7 25 5.0–5.6 50 4.2–4.9 75 3.4–4.1 100 <3.4 Scores were adjusted for medication usage by subtracting 20 points from the score (unless the score was 0).	x				
	Blood glucose	Nivel-PCD	FBG (mmol/L) or HbA1c (mmol/mol), measured by blood sample.	**Table:** Points Level 0 Diabetes (≥ 7 mmol/L) and HbA1c ≥86 10 Diabetes and HbA1c 75–85 20 Diabetes and HbA1c 64–74 30 Diabetes and HbA1c 54–63 40 Diabetes and HbA1c ≤53 60 No diabetes and FBG 6.1 ≤ 7.0 or non-FBG 7.8 ≤ 11.1 100 No diabetes and FBG <6.1 or non-FBG <7.8 Scores were adjusted for medication usage by subtracting 20 points from the score (unless the score was 0).	x				
	Blood pressure	Nivel-PCD	Systolic and diastolic blood-pressure (mmHg), measured by a calibrated sphygmomanometer within primary care.	**Table:** Points BP (mmHg) 0 ≥160 or ≥ 100 25 140–159 or 90–99 50 130–139 or 80–89 75 120–129 or < 80 100 <120/<80 Scores were adjusted for medication usage by subtracting 20 points from the score (unless the score was 0).	x				

Abbreviations: CBS, Statistics Netherlands; SQUASH, Short Questionnaire to Assess Health-enhancing Physical Activity; NDS, nicotine-delivery system; BMI, Body Mass Index; kg/m^2^, kilograms per square meter; Nivel, Netherlands Institute for Health Services Research; PCD, primary care database; non-HDL, Non-High-Density-Lipoprotein; mmol/L, millimoles per litre; FBG, Fasting Blood Glucose; HbA1c, haemoglobin A1c; mmol/mol, millimoles per mol; mmHg, millimetres of mercury.

#### 2.3.2 Sociodemographic characteristics

Scores were stratified by biological sex (women, men), and when the data allowed, also by age, education level, standardised yearly household income, and migration background. Age was categorised into 18–19, 80+, and the ages between 20 and 79 were categorised into 10-year strata. Following the CBS-classification, education was categorised into primary/pre-vocational education, secondary/vocational education, and tertiary/academic education; income status was classified into standardised household income quintiles relative to the Dutch population (in 2021); and migration background was defined using the birth country of the individual and their parents. For individuals born in the Netherlands and with both parents born in the Netherlands, the migration background was classified as ‘the Netherlands’. Individuals born abroad were classified according to their country of birth, and those born in the Netherlands with at least one parent born abroad were classified by the parents' birth countries, prioritising the mother's country. This was grouped into 10 categories based on the CBS-categorisation (see Table 2b). Due to privacy reasons (*n* < 10), the ‘Moroccan’ group and ‘other Africa’ were merged.

#### 2.3.3 LE8 score

The study outcome was the CVH of the Dutch adult population based on the LE8 score, total and stratified by biological sex. Mean scores were calculated for the individual LE8 metrics, the composite health factors, the composite behaviour factors, and the overall LE8 score. The scores ranged from 0 to 100; a score ≥ 80 was classified as ideal CVH, a score between 50 and 79 as intermediate CVH, and a score < 50 as poor CVH (Lloyd-Jones et al., 2022a).

### 2.4 Statistical analysis

Descriptive analyses were performed. Categorical variables were presented using frequencies (n) and percentages (%), and continuous variables were presented using the mean and standard deviation (SD).

#### 2.4.1 Nivel: blood lipids, blood glucose, and blood pressure

Laboratory data were not available for the complete Nivel study population (21.2% for lipids, 26.5% for glucose, 24.8% for blood pressure). Therefore, we categorised the available data into four groups based on the combination of registered ICPC-1 diagnosis and laboratory data: yes/yes, yes/no, no/yes, no/no. In Supplementary Table 1 the four created groups are presented.

For each metric, the four groups were evaluated separately and scored according to the scoring system presented in Table 1. The scores for the group representing the general population (group 4, Supplementary Table 1) were incremented by one score class relative to the standard scoring as presented in Table 1, to account for an overrepresentation of individuals with underlying health conditions within primary care data. The scores of the four groups were weighted based on their proportion in the overall study population and summed up to obtain a total score per LE8 metric. These metric scores were age-standardised using the 2022 Dutch population as the reference distribution to provide an estimate representative of the national population.

#### 2.4.2 CBS: BMI, diet, nicotine exposure, physical activity, and sleep

Individual metric scores for each LE8 metric were calculated following the scoring system described in Table 1. Individuals with missing data for a specific metric were excluded from that metric score. Extreme physical activity values were excluded (>6000 min, *n* = 31, 0.39%), and individuals with missing data for inhaled nicotine-delivery system usage were assumed to be non-smokers. According to the 2022 population distribution, age-standardised scores were calculated per metric. Individual metric scores were stratified by biological sex, age, migration background, income, and education, to provide insights into variation across sociodemographic groups.

Additionally, a complete case analysis was conducted for the behaviour factors. The mean score of the four behaviour factors was calculated and categorised into the three CVH score groups. The percentage of individuals within each subgroup was calculated, and the groups were stratified by biological sex, age, migration background, income, and education.

#### 2.4.3 LE8 score

The health factors composite score was calculated by adding the four mean metric scores together and dividing them by four. The same calculation was performed for the behaviour factors. The composite LE8 score was calculated by taking the mean of the eight metric mean scores, with each variable weighted equally. We presented the overall LE8 score for the total sample and stratified by biological sex.

All analyses were performed using R software (version 4.5.0).

### 2.5 Ethical compliance

Ethical approval was not required for this study because it used secondary data from national registries and statistical databases without access to directly identifiable personal information. Pseudonymised CBS-microdata were accessed and analysed exclusively within the secure CBS-environment, and anonymised Nivel data were provided in aggregated form. The applicable ethical and data governance considerations are described in the Ethics Declaration.

## 3 Results

### 3.1 Baseline characteristics

The Nivel study population consisted of 1,222,757 adults (18+). The mean age was 49.6 ± 18.5 years and 51% were women. The CBS study population consisted of 8027 adults (18+). The mean age was 51.9 ± 18.6 years and 52% were women. Characteristics of the study population are presented in Table 2a, Table 2b.

**Table 2a:** Study population characteristics (Dutch adults 18+) of the Life's Essential 8 factors blood lipids, blood glucose, and blood pressure retrieved from Nivel-PCD (2022).

	Total	Women	Men
Population, n (%)	1,222,757 (100.0)	623,503 (51.0)	599,254 (49.0)
Age in years, mean ± SD	49.6 ± 18.5	49.9 ± 18.8	49.1 ± 18.3
Blood pressure measurement, n (%)	303,755 (24.8)	161,262 (25.9)	142,493 (23.8)
<120/<80 mmHg	10.7%	12.2%	9.0%
120–129/<80 mmHg	10.8%	10.7%	10.8%
130–139/80–89 mmHg	32.8%	32.5%	33.2%
140–159/90–99 mmHg	34.4%	33.2%	35.8%
≥160/≥100 mmHg	11.3%	11.4%	11.2%
Blood pressure lowering medication^a^, n (%)	157,043 (12.8)	81,566 (13.1)	75,477 (12.6)
Diagnosis hypertension^b^, n (%)	248,413 (20.3)	133,161 (21.4)	115,252 (19.2)
Cholesterol measurement, n (%)	259,513 (21.2)	133,777 (21.5)	125,736 (21.0)
nHDL <3,4 mmol/L	48.0%	44.4%	51.7%
nHDL 3,4–4,1 mmol/L	27.3%	28.7%	25.6%
nHDL 4,2–4,9 mmol/L	15.7%	16.8%	14.5%
nHDL 5,0–5,6 mmol/L	6.4%	7.1%	5.8%
nHDL >5,6 mmol/L	2.7%	3.1%	2.4%
Cholesterol controlling medication^c^, n (%)	84,728 (6.7)	38,595 (6.2)	46,133 (7.7)
Diagnosis lipid metabolism disorder^d^, n (%)	135,011 (11.0)	68,385 (11.0)	66,626 (11.1)
Blood glucose measurement, n (%)	324,067 (26.5)	175,846 (28.2)	148,221 (24.7)
No diabetes + FBG <6.1 or non-FBG <7.8 mmol/L	40.8%	42.6%	38.7%
No diabetes + FBG 6.1–7.0 or non-FBG 7.8–11 mmol/L	35.6%	37.4%	33.4%
Diabetes (≥ 7 mmol/L) + HbA1c ≤53 mmol/mol	11.9%	10.2%	14.0%
Diabetes (≥ 7 mmol/L) + HbA1c 54–63 mmol/mol	6.1%	5.2%	7.1%
Diabetes (≥ 7 mmol/L) + HbA1c 64–74 mmol/mol	2.6%	2.2%	3.1%
Diabetes (≥ 7 mmol/L) + HbA1c 75–85 mmol/mol	0.9%	0.8%	1.2%
Diabetes (≥ 7 mmol/L) + HbA1c ≥86 mmol/mol	0.7%	0.6%	0.9%
Insulin medication, n (%)	12,131 (1.0)	6161 (1.0)	5970 (1.0)
Diagnosis diabetes mellitus^e^, n (%)	97,907 (8.0)	44,931 (7.2)	52,976 (8.8)

Abbreviations: Nivel, Netherlands Institute for Health Services Research; PCD, primary care database; n, number; SD, standard deviation; mmHg, millimetres of mercury; ICPC-1, international classification of primary care; HDL, Non-High-Density Lipoprotein; mmol/L, millimoles per litre; FBG, Fasting blood Glucose; HbA1c, haemoglobin A1c; mmol/mol, millimoles per mol.

a = ATC C02AC01, C02DC01, C02DD01, C03, C07, C08, C09.

b = ICPC-1 k85 (elevated blood pressure without hypertension), k86 (uncomplicated hypertension), k87 (hypertension with involvement target organs).

c = ATC C10.

d = ICPC-1 T93 (lipid metabolism disorder).

e = ICPC-1 T90 (diabetes mellitus).

**Table 2b:** Study population characteristics (Dutch adults 18+) of the Life's Essential 8 metrics BMI, physical activity, diet, nicotine exposure, sleep, retrieved from Statistics Netherlands (2022).

	Total	Women	Men
Population, n (%)	8027 (100.0)	4162 (51.9)	3865 (48.1)
Age in years, mean ± SD	51.9 ± 18.6	51.7 ± 18.8	52.1 ± 18.4
Migration background^a^, n (%)			
The Netherlands	6349 (79.1)	3269 (78.5)	3080 (79.7)
Caribbean Netherlands	62 (0.8)	32 (0.8)	30 (0.8)
Indonesia	234 (2.9)	124 (3.0)	110 (2.8)
Marocco	89 (1.1)	43 (1.0)	46 (1.2)
Suriname	137 (1.7)	85 (2.0)	52 (1.3)
Türkiye	138 (1.7)	54 (1.3)	84 (2.2)
Other Africa	95 (1.2)	49 (1.2)	46 (1.2)
Other Europa	591 (7.4)	333 (8.0)	258 (6.7)
Other America and Oceania	103 (1.3)	53 (1.3)	50 (1.3)
Other Asia	229 (2.9)	120 (2.9)	109 (2.8)
Education level^b^, n (%)			
Primary education and pre-vocational education	1911 (24.2)	1080 (26.4)	831 (21.9)
Vocational and secondary education	2904 (36.8)	1400 (34.2)	1504 (39.6)
Tertiary and academic education	3082 (39.0)	1617 (39.5)	1465 (38.6)
Standardised yearly household income^c^, n (%)			
First quintile (≤ €19.800)	915 (11.6)	509 (12.4)	406 (10.7)
Second quintile (€19.801–€26.400)	1324 (16.8)	762 (18.6)	562 (14.8)
Third quintile (€26.401–€33.700)	1624 (20.6)	816 (19.9)	808 (21.3)
Fourth quintile (€33.701–€43.200)	1907 (24.1)	955 (23.3)	952 (25.0)
Fifth quintile (> €43.200)	2129 (27.0)	1056 (25.8)	1073 (28.2)
Diet categories, n (%)			
≥250 g fruits/day	948 (11.9)	481 (11.6)	467 (12.2)
≥200 g vegetables/day	1100 (13.8)	629 (15.1)	471 (12.3)
≤ 1 glass alcohol/day	7267 (90.9)	3990 (96.1)	3277 (85.3)
≥1 a week fish	4485 (56.1)	2371 (57.1)	2114 (55.0)
Met diet categories, n (%)			
0 food categories	281 (3.5)	57 (1.4)	224 (5.8)
1 food category	2960 (37.0)	1479 (35.6)	1481 (38.5)
2 food categories	3560 (44.5)	1935 (46.6)	1625 (42.3)
3 food categories	1052 (13.2)	602 (14.5)	450 (11.7)
4 food categories	141 (1.8)	79 (1.9)	62 (1.6)
Physical Activity, n (%)			
0 min/week	608 (8.4)	397 (10.6)	211 (6.0)
1–30 min/week	39 (0.5)	23 (0.6)	16 (0.5)
30–60 min/week	78 (1.1)	41 (1.1)	37 (1.0)
60–90 min/week	185 (2.5)	102 (2.7)	83 (2.3)
90–12- min/week	92 (1.3)	49 (1.3)	43 (1.2)
120–150 min/week	239 (3.3)	139 (3.7)	100 (2.8)
≥150 min/week	6032 (82.9)	2981 (79.9)	3051 (86.2)
Sleeping problems, n (%)			
Not at all	3154 (39.3)	1364 (32.8)	1790 (46.3)
A little	2854 (35.6)	1572 (37.8)	1282 (33.2)
Quite	1039 (12.9)	629 (15.1)	410 (10.6)
A lot	638 (7.9)	383 (9.2)	255 (6.6)
Quite a lot	341 (4.2)	213 (5.1)	128 (3.3)
Nicotine exposure, n (%)			
Never-smokers	3800 (47.3)	2155 (51.8)	1645 (42.6)
Current smokers	1356 (16.9)	582 (14.0)	774 (20.0)
Former smoking	2870 (35.8)	1425 (34.2)	1445 (37.4)
Living with active indoor smoker	1616 (20.1)	760 (18.3)	856 (22.1)
Inhaled nicotine-delivery systems	191 (2.4)	94 (2.3)	98 (2.5)
BMI (kg/m^2^), n (%)			
<18.5	145 (1.8)	103 (2.5)	42 (1.1)
<25	3743 (47.1)	2091 (50.9)	1652 (43.0)
<30	2860 (36.0)	1258 (30.6)	1602 (41.7)
<35	869 (10.9)	452 (11.0)	417 (10.9)
<40	233 (2.9)	145 (3.5)	88 (2.3)
≥ 40	95 (1.2)	56 (1.4)	39 (1.0)

Abbreviations: n, number; SD, standard deviation; BMI, Body Mass Index; min/week, minutes per week; kg, kilograms; m^2^, squared meters.

a Combination of country of birth individual and parents.

b Until age 25 highest followed, above 25 highest achievements.

c Based on the standardised yearly household income 2021.

### 3.2 LE8 score

In Fig. 1 and Supplementary Table 2, the mean LE8 scores are shown. The mean LE8 score was 73.0 out of 100, corresponding to intermediate CVH. The composite behaviour factors score was lower than the composite health factors score (69.4 versus 76.5, both intermediate CVH). Physical activity was the highest scoring metric (90.5 (ideal CVH)) and diet the lowest (42.8 (poor CVH)). For the overall LE8 score, women scored higher than men (73.5 versus 72.3, both intermediate CVH). Except for physical activity and sleep, women scored higher on all individual LE8 metrics.

**Fig. 1:**
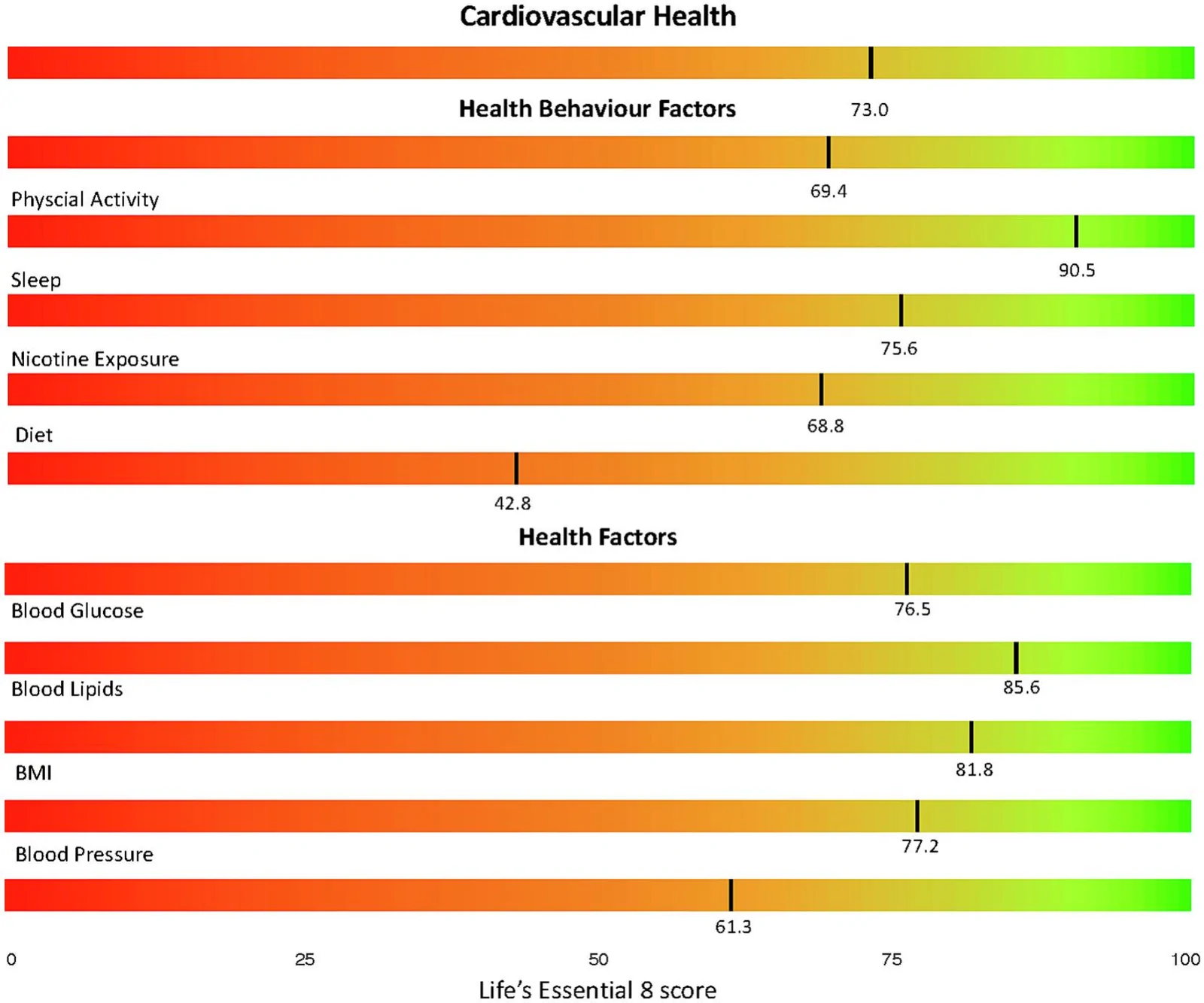
Cardiovascular health of the Dutch adult's population in 2022, based on Life's Essential 8 framework. Heat scales, ranging from 0 to 100, displaying the Life's Essential 8 scores in the Dutch adult population. The mean score is displayed using a bold black line. Abbreviation: BMI, Body Mass Index.

Supplementary Tables 3–7 shows the individual scores of the LE8 metrics BMI, diet, nicotine exposure, physical activity, sleep, stratified by sociodemographic characteristics. For all five metrics, individuals with a tertiary/academic education scored higher than individuals with primary/pre-vocational education, for both, women and men. The same applied for income; individuals with the highest incomes had higher scores than individuals with the lowest incomes. Scores declined with age for BMI and physical activity, while increased with age for diet and nicotine exposure; no pattern was observed for sleep. Scores for sleep and physical activity were the lowest for ‘Marrocco’ (62.7 and 76.7), and the highest for ‘other Africa’ (77.9 and 89.9). Scores for nicotine exposure and diet were the highest for ‘Marrocco’ (78.2 and 48.0), and the lowest for ‘Türkiye’ (53.7) and ‘other America and Oceania’ (38.6). Scores for BMI were the lowest for ‘Caribbean Netherlands’ (66.9) and highest for ‘other Asia’ (81.0).

Fig. 2 and Supplementary Table 8 present the distribution of individuals with complete data on all behaviour factors (*n* = 6967) across ideal, intermediate, and poor CVH groups, stratified by sociodemographic characteristics. Most individuals had an intermediate CVH (56.8%; *n* = 3958), followed by an ideal CVH (32.9%; *n* = 2294) and a poor CVH (10.3%; *n* = 715). The percentages of ideal, intermediate and poor CVH were comparable among women and men. Adults aged 80+ years and 20–29 years had the highest percentage of poor CVH (respectively, 17.2% and 14.2%). The percentage of individuals with an ideal CVH increased with education and income level (23.1 to 42.8% and 21.3 to 40.5%, respectively), and the percentage of individuals with a poor CVH decreased with education and income level (16.5 to 5.7% and 22.9 to 5.4%, respectively). Individuals with a migration background ‘Caribbean Netherlands’ and ‘Türkiye’ had the highest percentage of low CVH (20 and 23%). Individuals with a migration background ‘Africa’ had highest percentage of ideal CVH (39.1%).

**Fig. 2:**
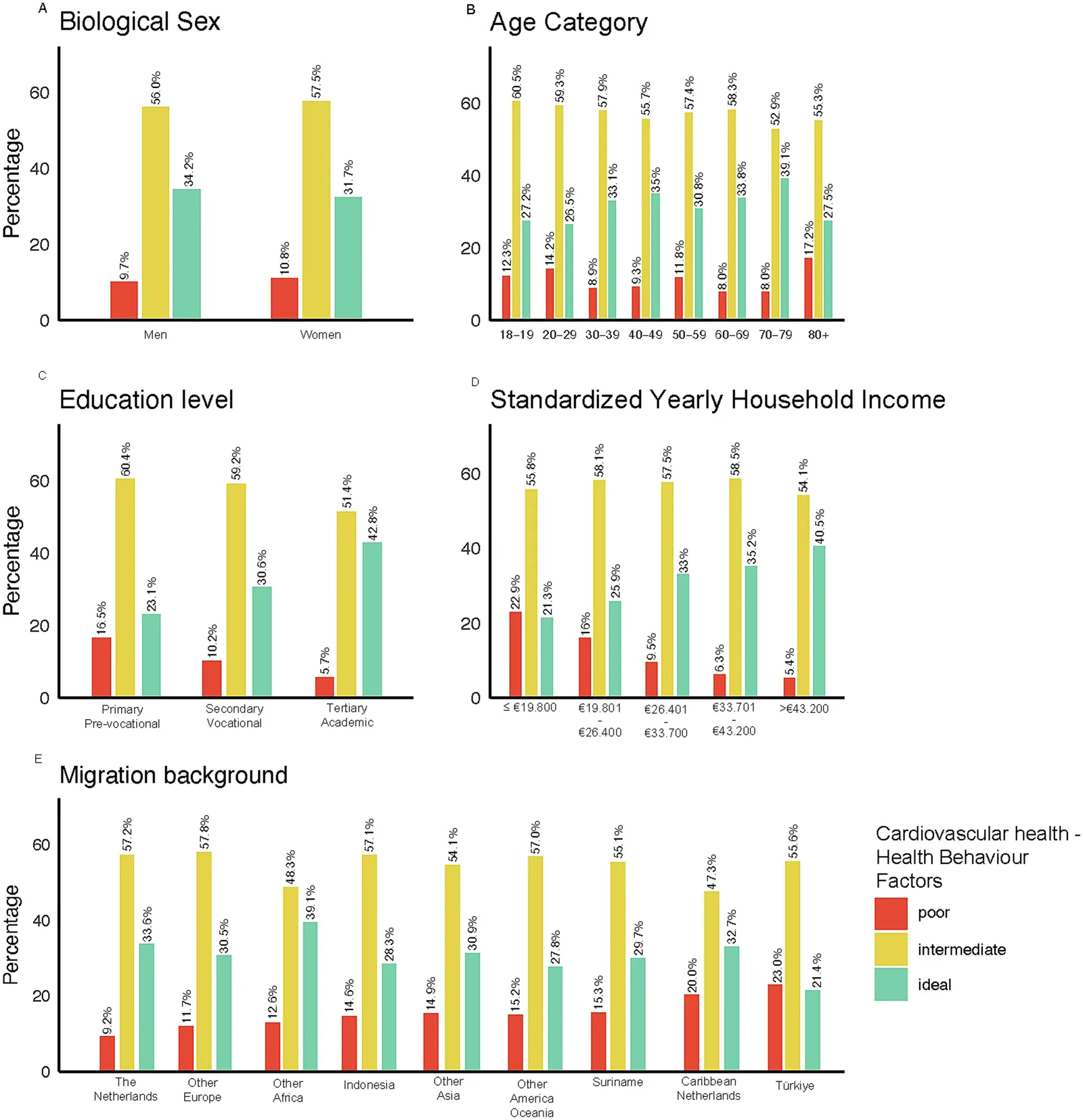
Cardiovascular health levels among adults in the Netherlands across five sociodemographic characteristics based on Life's Essential 8 behaviour factors, in 2022. Five bar graphs showing the percentage of individuals with an ideal, intermediate, and poor cardiovascular health (based on the Life's Essential 8 behaviour factors) across sociodemographic characteristics. (A) shows the distribution within biological sex, (B) within age categories, (C) within education level, (D) within income and (E) within migration background.

## 4 Discussion

The intermediate CVH with a mean LE8 score of 73.0 aligns with findings from two Dutch cohort studies (LE8 score 69.8 and 72.3) (van Loon et al., 2025; Spronk et al., 2025), while exceeding those reported in international studies. The US reported a mean LE8 score of 64.7 (Lloyd-Jones et al., 2022b), France 66 (Deraz et al., 2025), Spain 66.3 (Hernández-Martínez et al., 2024), and the United Kingdom (UK) 69.5 (Zhang et al., 2023). While differences between countries can partly be explained by demographic and health system differences, comparison requires caution. The LE8 framework made cross-country comparison easier, but studies slightly adjusted the LE8 metric quantification to match national data, reducing comparability.

The variation between the individual LE8 scores is well documented in the literature (Deraz et al., 2025; Lloyd-Jones et al., 2022b; Hernández-Martínez et al., 2024). Like our study, diet was often the lowest LE8 metric score and a major contributor to a reduced LE8 CVH (López-Bueno et al., 2024). Besides diet, blood pressure scored low in our study (mean 61.0). Hypertension is one of the most important risk factors of the global prevalence of LE8, with a pooled prevalence of 29.4% (López-Bueno et al., 2024). Which is also in line with data on the Global Burden of Disease in the Netherlands: high systolic blood pressure is a big contributor to CVD deaths and DALYs (Institute for Health Metrics and Evaluation, 2023). Therefore, besides the emphasis on improving diet to improve CVH, blood pressure is an important factor for CVD prevention.

In contrast, physical activity was the highest LE8 metric score (mean 90.5); a score similar to the previously mentioned Dutch cohort study (van Loon et al., 2025), but is significantly higher than scores in the US (mean 51.6) (Lloyd-Jones et al., 2022b), Spain (mean 60.3) (Hernández-Martínez et al., 2024), France (mean 65.2) (Deraz et al., 2025), and the UK (mean 76.9) (Zhang et al., 2023). While the scaling of moderate or vigorous activities may account for part of the variation, the Dutch population is known to be more active due to the cycling and walking culture and supportive infrastructure for these activities in the country (Fishman et al., 2015).

The heterogeneity in LE8 score between sociodemographic characteristics underscores the importance of stratifying analyses. Stratification by socioeconomic position revealed that groups with lower incomes and primary education levels had lower LE8 scores, which is a well-known but persistent disparity (Janković et al., 2021). Socioeconomic position is a key determinant in CVH and is associated with a higher prevalence of cardiovascular risk factors and higher CVD incidence and mortality (Janković et al., 2021; Wang et al., 2024). Stratification by biological sex revealed smaller sex differences in our study relative to other studies, but consistent higher scores for women compared to men (Peng et al., 2018). Within the individual LE8 metrics, we observed, for example, more than 7 points difference in the scores of nicotine exposure. While the sex difference in smoking is becoming smaller over time, the patterns still apply, and the prevalence of smoking among men remains higher compared to women, which can explain the difference (Janssen and van Poppel, 2015). Stratification by migration background revealed score differences within the population. Individuals with an African background had the highest proportion of ideal CVH and individuals with a Turkish migration background showed the highest proportion of poor CVH. Further stratification can reveal more heterogeneity, which is seen in another study where Ghanaian men had a lower fatal CVD risk and African Surinamese and Turkish men showed a higher fatal and non-fatal CVD risk (Perini et al., 2018). Therefore, while stratification can reveal subgroup differences, heterogeneity also underlined the importance of personalised care (van der Leeuw et al., 2014).

This study highlights several implications for improving CVH in the Netherlands, with a particular focus on diet, blood pressure, and individuals with lower income and primary-level education. While improving CVH is complex because it often requires behaviour change, implementing interventions at multiple levels can support sustainable improvement (Moise et al., 2022). At the policy level, subsidies for fruits and vegetables may encourage healthier choices (Green et al., 2013). At the community level, encouraging community engagement can tailor interventions to specific needs (Diab et al., 2023). Lastly, the individual level is essential, while improving population health is a national initiative, research and interventions must account for heterogeneity in the population (Moise et al., 2022; Diaz et al., 2021).

This study has several strengths and limitations. Combining two datasets strengthens the findings but also introduces certain limitations. First, aggregated Nivel data limited stratification by sociodemographic characteristics, so stratified analyses were restricted to the behaviour factors (CBS data). Since our results indicated clear heterogeneity, obtaining individual-level health data is needed to stratify health factors and capture the diversity within these factors. Second, CBS data were self-reported, which might have influenced accuracy, as people tend to report socially desirable answers (Prince et al., 2008). Third, Nivel data relied on individuals registered to primary health care practices limiting the ability to represent individuals in the broader population who remain undiagnosed or untreated for risk factors such as hypertension, dyslipidaemia, or diabetes. While a solution for better national data coverage might be to collect lifestyle data together with health data, in EHR (Lloyd-Jones et al., 2022a), the last limitation will remain.

Despite these limitations, a considerable strength of this study is that both sources are suitable for long-term trend measurement, which creates opportunities to monitor and increase knowledge on CVH development over time, resulting in better identification of areas for improvement (Sebastian et al., 2025; Radovanovic et al., 2023). This study provides a baseline to build upon in further research.

## 5 Conclusions

CVH in the Netherlands is intermediate with an LE8 score of 73.0/100. Heterogeneity in scores were observed between sociodemographic characteristics, which identifies the need of stratification and provides information to tailor interventions.

## Declaration of generative AI use

During the preparation of this work AI tools were used for language editing to improve readability and grammar. Besides, AI was used to assist in generating example code structures and debugging R code. All AI content was reviewed, validated, and edited. The authors take full responsibility for the content of the published article.

## CRediT authorship contribution statement

**Lieke M. Steendam:** Writing – review & editing, Writing – original draft, Visualization, Software, Project administration, Methodology, Formal analysis, Data curation, Conceptualization. **Ilonca Vaartjes:** Writing – review & editing, Supervision, Project administration, Methodology, Funding acquisition, Conceptualization. **Raymond H. Wimmers:** Writing – review & editing, Resources, Methodology, Funding acquisition, Conceptualization. **Yvonne Koop:** Writing – review & editing, Visualization, Software, Project administration, Methodology, Funding acquisition, Formal analysis, Data curation, Conceptualization. **Dutch Heart Foundation Cardiovascular Health Working Group consisting of Michiel L. Bots, Monika Hollander, Janet M. Kist, Fabrice M.A.C. Martens, Jeanine E. Roeters van Lennep:** Conceptualization, Methodology, Writing – review & editing and **Marianne J. Heins:** Data curation, Resources, Writing – review & editing. All authors gave their final approval.

## Funding

This study was supported by grants from the 10.13039/100002129Dutch Heart Foundation (04-003-2024-0161).

## Declaration of competing interest

The authors declare that they have no known competing financial interests or personal relationships that could have appeared to influence the work reported in this paper.

## Figures and Tables

**Table:** 

Michiel L. Bots	Epidemiologist	Julius Center for Health Sciences and Primary Care, University Medical Centre Utrecht, Utrecht University, Utrecht, The Netherlands
Marianne J. Heins	Researcher	Nivel, Utrecht, The Netherlands
Monika Hollander	Epidemiologist General Practitioner	Department of General Practice, Amsterdam UMC-location VUmc, Amsterdam Public Health Research Institute, Amsterdam, The Netherlands.
Janet M. Kist	Epidemiologist General Practitioner	Health Campus The Hague & Public Health and Primary Care, Leiden University Medical Center, Leiden, The Netherlands
Fabrice M.A.C. Martens	Medical specialist - cardiology	Department of Cardiology, Amsterdam University Medical Center, Amsterdam, The Netherlands.
Jeanine E. Roeters van Lennep	Medical specialist – internal medicine	Department of Internal Medicine, Erasmus MC Cardiovascular Institute Erasmus MC University Medical Center, Rotterdam, Netherlands

## Data Availability

Data are not publicly available; access can be requested from Nivel and CBS.

## Appendix B Dutch Heart Foundation Cardiovascular Health Working Group

Individual authors:
