# Supplementary material for: Cardiovascular health based on the life's essential 8: overall status and subgroup differences across sociodemographic characteristics in the Netherlands
Source: Prev Med Rep. 2026 Oct 23;71:103674. doi: 10.1016/j.pmedr.2026.103674 (PMC13760061; doi:10.1016/j.pmedr.2026.103674)
Supplement: Supplementary file 1 [file mmc1.docx]

# Appendices

**Supplement Table 1.** Distribution of the four created analysis groups for Nivel-PCD

|  | **Blood pressure** | | | **Blood lipids** | | | **Blood glucose** | | |
| --- | --- | --- | --- | --- | --- | --- | --- | --- | --- |
|  | **Total** | **Women** | **Men** | **Total** | **Women** | **Men** | **Total** | **Women** | **Men** |
| **Population groups, n (%)** | 1,222,757 (100.0) | 623,503 (51.0) | 599,254 (49.0) | 1,222,757 (100.0) | 623,503 (51.0) | 599,254 (49.0) | 1,222,757 (100.0) | 623,503 (51.0) | 599,254 (49.0) |
| Group 1: ICPC-1 + Measurement | 13.4% | 14.3% | 12.4% | 6,2% | 6.3% | 6.1% | 5.9% | 5.4% | 6.5% |
| Group 2: ICPC-1 only | 6.9% | 7.0% | 6.8% | 4,8% | 4.6% | 5.0% | 2.1% | 1.9% | 2.3% |
| Group 3: measurement only | 11.5% | 11.6% | 11.4% | 15,0% | 15.1% | 14.9% | 20.6% | 22.9% | 18.2% |
| Group 4: ‘general population’ | 68.2% | 67.1% | 69.4% | 74,0% | 73.9% | 74.0% | 71.4% | 69.9% | 73.0% |

*Abbreviations:* *Nivel-PCD, Nivel Primary Care Database; n, number;* ICPC-1, international classification of primary care;

*‘general population’ = defined as no ICPC-1 diagnosis nor laboratory measurement*

**Supplement Table 2.** Mean Life’s Essential 8 scores of the Dutch adult population, weighted by the 2022 population age-distribution

|  |  | **Total** |  | **Women** |  | **Men** |  |
| --- | --- | --- | --- | --- | --- | --- | --- |
|  |  | **Mean (95% CI)** | **n** | **Mean (95% CI)** | **n** | **Mean (95% CI)** | **n** |
| **Health behaviour factors** | | **69.4** |  | **69.5** |  | **69.3** |  |
|  | Sleep health | 75.6 | 8,026 | 72.3 | 4,161 | 79.1 | 3,865 |
|  | Physical activity | 90.5 | 7,273 | 88.6 | 3,732 | 92.4 | 3,541 |
|  | Diet | 42.8 | 7,994 | 44.8 | 4,152 | 40.6 | 3,842 |
|  | Nicotine exposure | 68.8 | 7,735 | 72.4 | 4,047 | 65.0 | 3,688 |
| **Health Factors** | | **76.5** |  | **77.4** |  | **75.2** |  |
|  | BMI | 77.2 | 7,945 | 77.9 | 4,105 | 76.5 | 3,840 |
|  | Blood pressure | 61.3 | 1,222,757 | 62.7 | 623,503 | 59.3 | 599,254 |
|  | Blood lipids | 81.8 | 1,222,757 | 82.5 | 623,503 | 80.7 | 599,254 |
|  | Blood glucose | 85.6 | 1,222,757 | 86.6 | 623,503 | 84.4 | 599,254 |
|  | **Total LE8 score** | **73.0** |  | **73.5** |  | **72.3** |  |

*Abbreviations:* n, number; BMI, Body Mass index; LE8, Life’s Essential 8

**Supplement Table 3.** Mean scores of the Life’s Essential 8 metric BMI for the Dutch adult population among sociodemographic characteristics

|  | | **Total** | | **Women** | | **Men** | |
| --- | --- | --- | --- | --- | --- | --- | --- |
|  | | **Mean** | **n, %** | **Mean** | **n, %** | **Mean** | **n, %** |
| **Sample** | |  |  |  |  |  |  |
| **Age in years** | |  |  |  |  |  |  |
|  | 18-19 | 89.1 | 233 (2.9) | 88.2 | 123 (3.0) | 90.1 | 110 (2.9) |
|  | 20-29 | 84.7 | 979 (12.3) | 83.4 | 531 (12.9) | 86.3 | 448 (11.7) |
|  | 30-39 | 79.7 | 1,132 (14.2) | 79.2 | 567 (13.8) | 80.2 | 565 (14.7) |
|  | 40-49 | 76.2 | 1,150 (14.5) | 77.2 | 603 (14.7) | 75.1 | 547 (14.2) |
|  | 50-59 | 73.4 | 1,377 (17.3) | 75.0 | 733 (17.9) | 71.6 | 644 (16.8) |
|  | 60-69 | 74.3 | 1,450 (18.3) | 76.0 | 733 (17.9) | 72.6 | 717 (18.7) |
|  | 70-79 | 75.2 | 1,172 (14.8) | 75.5 | 561 (13.7) | 75.0 | 611 (15.9) |
|  | 80+ | 78.9 | 452 (5.7) | 80.6 | 254 (6.2) | 76.7 | 198 (5.2) |
| **Migration background country^** | |  |  |  |  |  |  |
|  | Caribbean Netherlands | 66.9 | 60 (0.8) | 62.4 | 31 (0.8) | 71.7 | 29 (0.8) |
|  | Other Europa | 77.7 | 584 (7.4) | 78.0 | 328 (8.0) | 77.2 | 256 (6.7) |
|  | Indonesia | 78.9 | 231 (2.9) | 82.6 | 122 (3.0) | 74.8 | 109 (2.8) |
|  | Marocco | 72.8 | 86 (1.1) | 71.1 | 42 (1.0) | 74.5 | 44 (1.1) |
|  | The Netherlands | 77.5 | 6,292 (79.2) | 78.1 | 3,228 (78.6) | 76.9 | 3,064 (79.8) |
|  | Other Africa | 77.6 | 93 (1.2) | 74.0 | 47 (1.1) | 81.2 | 46 (1.2) |
|  | Other America and Oceania | 71.9 | 102 (1.3) | 76.3 | 52 (1.3) | 67.3 | 50 (1.3) |
|  | Other Asia | 81.0 | 228 (2.9) | 82.2 | 119 (2.9) | 79.6 | 109 (2.8) |
|  | Suriname | 77.0 | 134 (1.7) | 76.6 | 83 (2.0) | 77.6 | 51 (1.3) |
|  | Türkiye | 68.0 | 135 (1.7) | 72.3 | 53 (1.3) | 65.2 | 82 (2.1) |
| **Education level*** | |  |  |  |  |  |  |
|  | Primary and pre-vocational education | 71.1 | 1881 (24.1) | 71.3 | 1062 (26.3) | 70.8 | 819 (21.7) |
|  | Vocational and secondary education | 75.4 | 2879 (36.8) | 75.9 | 1380 (34.1) | 75.0 | 1499 (39.7) |
|  | Tertiary and academic education | 83.0 | 3056 (39.1) | 84.3 | 1599 (39.6) | 81.7 | 1457 (38.6) |
| **Standardized yearly household income**± | |  |  |  |  |  |  |
|  | First quintile (≤ €19.800) | 74.6 | 890 (11.4) | 74.7 | 495 (12.2) | 74.5 | 395 (10.5) |
|  | Second quintile (€19.801-€26.400) | 73.3 | 1305 (34.6) | 73.8 | 747 (18.5) | 72.5 | 558 (14.8) |
|  | Third quintile (€26.401-€33.700) | 76.4 | 1612 (42.7) | 77.1 | 808 (20.0) | 75.8 | 804 (21.3) |
|  | Fourth quintile (€33.701-€43.200) | 78.3 | 1897 (50.2) | 78.5 | 948 (23.5) | 78.1 | 949 (25.1) |
|  | Fifth quintile (> €43.200) | 80.5 | 2115 (56.0) | 82.4 | 1044 (25.8) | 78.7 | 1071 (28.4) |

* Until age 25 highest followed, above 25 highest achievements

^ combination of country of birth individual and parents

± based on the standardized yearly household income 2021

*Abbreviations:* BMI, Body Mass index; n, number

**Supplement Table 4.** Mean scores of the Life’s Essential 8 metric diet for the Dutch adult population among sociodemographic characteristics

|  | | **Total** | | **Women** | | **Men** | |
| --- | --- | --- | --- | --- | --- | --- | --- |
|  | | **Mean** | **n, %** | **Mean** | **n, %** | **Mean** | **n, %** |
| **Sample** | |  |  |  |  |  |  |
| **Age in years** | |  |  |  |  |  |  |
|  | 18-19 | 33.3 | 235 (2.9) | 36.0 | 125 (3.0) | 30.2 | 110 (2.9) |
|  | 20-29 | 36.0 | 991 (12.4) | 37.9 | 538 (13.0) | 33.8 | 453 (11.8) |
|  | 30-39 | 40.2 | 1,137 (14.2) | 42.8 | 576 (13.9) | 37.6 | 561 (14.6) |
|  | 40-49 | 42.2 | 1,155 (14.4) | 44.2 | 607 (14.6) | 40.0 | 548 (14.3) |
|  | 50-59 | 42.7 | 1,380 (17.3) | 44.9 | 739 (17.8) | 40.1 | 641 (16.7) |
|  | 60-69 | 47.2 | 1,456 (18.2) | 49.4 | 739 (17.8) | 44.9 | 717 (18.7) |
|  | 70-79 | 49.2 | 1,181 (14.8) | 50.4 | 567 (13.7) | 48.2 | 614 (16.0) |
|  | 80+ | 46.5 | 459 (5.7) | 46.8 | 261 (6.3) | 46.1 | 198 (5.2) |
| **Migration background country^** | |  |  |  |  |  |  |
|  | Caribbean Netherlands & Kingdom of the Netherlands | 42.6 | 61 (0.8) | 43.8 | 32 (0.8) | 41.4 | 29 (0.8) |
|  | Other Europa | 46.0 | 589 (7.4) | 47.6 | 332 (8.0) | 43.9 | 257 (6.7) |
|  | Indonesia | 46.6 | 232 (2.9) | 49.2 | 124 (3.0) | 43.5 | 108 (2.8) |
|  | Marocco | 48.0 | 89 (1.1) | 46.5 | 43 (1.0) | 49.5 | 46 (1.2) |
|  | The Netherlands | 42.8 | 6,331 (79.2) | 44.7 | 3,264 (78.6) | 40.8 | 3,067 (79.8) |
|  | Other Africa | 44.9 | 89 (1.1) | 44.7 | 47 (1.1) | 45.2 | 42 (1.1) |
|  | Other America and Oceania | 38.6 | 103 (1.3) | 41.0 | 53 (1.3) | 36.0 | 50 (1.3) |
|  | Other Asia | 43.9 | 225 (2.8) | 44.3 | 118 (2.8) | 43.5 | 107 (2.8) |
|  | Suriname | 45.8 | 137 (1.7) | 47.6 | 85 (2.0) | 42.8 | 52 (1.4) |
|  | Türkiye | 38.6 | 138 (1.7) | 39.8 | 54 (1.3) | 37.8 | 84 (2.2) |
| **Education level*** | |  |  |  |  |  |  |
|  | Primary and pre-vocational education | 41.4 | 1,897 (24.1) | 43.6 | 1,075 (26.3) | 38.6 | 822 (21.8) |
|  | Vocational and secondary education | 41.5 | 2,892 (36.8) | 43.5 | 1,397 (34.2) | 39.7 | 1,495 (39.6) |
|  | Tertiary and academic education | 45.7 | 3,076 (39.1) | 47.2 | 1,615 (39.5) | 44.2 | 1,461 (38.7) |
| **Standardized yearly household income**± | |  |  |  |  |  |  |
|  | First quintile (≤ €19.800) | 41.4 | 900 (11.4) | 42.4 | 506 (12.4) | 40.0 | 394 (10.4) |
|  | Second quintile (€19.801-€26.400) | 42.9 | 1,315 (16.7) | 44.0 | 756 (18.5) | 41.3 | 559 (14.8) |
|  | Third quintile (€26.401-€33.700) | 42.2 | 1,620 (20.6) | 43.8 | 816 (20.0) | 40.6 | 804 (21.3) |
|  | Fourth quintile (€33.701-€43.200) | 42.6 | 1,904 (24.2) | 44.7 | 954 (23.3) | 40.6 | 950 (25.1) |
|  | Fifth quintile (> €43.200) | 45.2 | 2,128 (27.0) | 48.0 | 1,056 (25.8) | 42.4 | 1,072 (28.4) |

* Until age 25 highest followed, above 25 highest achievements

^ combination of country of birth individual and parents

± based on the standardized yearly household income 2021

*Abbreviations:* n, number

**Supplement Table 5.** Mean scores of the Life’s Essential 8 metric nicotine exposure for the Dutch adult population among sociodemographic characteristics

|  | | **Total** | | **Women** | | **Men** | |
| --- | --- | --- | --- | --- | --- | --- | --- |
|  | | **Mean** | **n, %** | **Mean** | **n, %** | **Mean** | **n, %** |
| **Sample** | |  |  |  |  |  |  |
| **Age in years** | |  |  |  |  |  |  |
|  | 18-19 | 64.9 | 232 (3.0) | 64.2 | 124 (3.1) | 65.7 | 108 (2.9) |
|  | 20-29 | 62.5 | 978 (12.6) | 65.8 | 527 (13.0) | 58.7 | 451 (12.2) |
|  | 30-39 | 68.3 | 1,120 (14.5) | 74.8 | 565 (14.0) | 61.7 | 555 (15.0) |
|  | 40-49 | 69.5 | 1,125 (14.5) | 73.1 | 594 (14.7) | 65.5 | 531 (14.4) |
|  | 50-59 | 68.1 | 1,336 (17.3) | 71.9 | 719 (17.8) | 63.6 | 617 (16.7) |
|  | 60-69 | 69.8 | 1,398 (18.1) | 71.4 | 721 (17.8) | 68.1 | 677 (18.4) |
|  | 70-79 | 75.2 | 1,116 (14.4) | 78.4 | 547 (13.5) | 72.2 | 569 (15.4) |
|  | 80+ | 79.7 | 430 (5.6) | 83.8 | 250 (6.2) | 74.1 | 180 (4.9) |
| **Migration background country^** | |  |  |  |  |  |  |
|  | Caribbean Netherlands | 61.3 | 60 (0.8) | 67.4 | 31 (0.8) | 54.8 | 29 (0.8) |
|  | Other Europa | 65.8 | 573 (7.4) | 68.5 | 326 (8.1) | 62.3 | 247 (6.7) |
|  | Indonesia | 64.2 | 228 (2.9) | 68.3 | 121 (3.0) | 59.6 | 107 (2.9) |
|  | Marocco | 78.2 | 89 (1.2) | 89.9 | 43 (1.1) | 67.3 | 46 (1.2) |
|  | The Netherlands | 70.5 | 6,108 (79.0) | 73.4 | 3,177 (78.5) | 67.3 | 2,931 (79.5) |
|  | Other Africa | 75.9 | 93 (1.2) | 75.9 | 48 (1.2) | 75.8 | 45 (1.2) |
|  | Other America and Oceania | 68.3 | 96 (1.2) | 71.9 | 50 (1.2) | 64.5 | 46 (1.2) |
|  | Other Asia | 66.0 | 221 (2.9) | 75.4 | 116 (2.9) | 55.6 | 105 (2.8) |
|  | Suriname | 68.0 | 132 (1.7) | 75.0 | 82 (2.0) | 56.5 | 50 (1.4) |
|  | Türkiye | 53.7 | 135 (1.7) | 67.5 | 53 (1.3) | 44.9 | 82 (2.2) |
| **Education level*** | |  |  |  |  |  |  |
|  | Primary and pre-vocational education | 64.3 | 1,834 (24.1) | 69.0 | 1,038 (26.1) | 58.1 | 796 (21.9) |
|  | Vocational and secondary education | 66.3 | 2,812 (37.0) | 69.1 | 1,368 (34.4) | 63.6 | 1,444 (39.8) |
|  | Tertiary and academic education | 75.6 | 2,964 (38.9) | 78.7 | 1,576 (39.6) | 72.2 | 1,388 (38.3) |
| **Standardized yearly household income**± | |  |  |  |  |  |  |
|  | First quintile (≤ €19.800) | 60.1 | 891 (11.7) | 66.5 | 499 (12.5) | 52.0 | 392 (10.8) |
|  | Second quintile (€19.801-€26.400) | 66.9 | 1,286 (16.9) | 69.4 | 747 (18.7) | 63.6 | 539 (14.9) |
|  | Third quintile (€26.401-€33.700) | 69.1 | 1,573 (20.7) | 72.0 | 797 (20.0) | 66.0 | 776 (21.4) |
|  | Fourth quintile (€33.701-€43.200) | 70.3 | 1,829 (24.0) | 74.7 | 930 (23.3) | 65.8 | 899 (24.8) |
|  | Fifth quintile (> €43.200) | 75.6 | 2,035 (26.7) | 78.3 | 1,013 (25.4) | 72.8 | 1,022 (28.2) |

* Until age 25 highest followed, above 25 highest achievements

^ combination of country of birth individual and parents

± based on the standardized yearly household income 2021

*Abbreviations:* n, number

**Supplement Table 6.** Mean scores of the Life’s Essential 8 metric physical activity for the Dutch adult population among sociodemographic characteristics

|  | | **Total** | | **Women** | | **Men** | |
| --- | --- | --- | --- | --- | --- | --- | --- |
|  | | **Mean CVH** | **n, %** | **Mean CVH** | **n, %** | **Mean**  **CVH** | **n, %** |
| **Sample** | |  |  |  |  |  |  |
| **Age (in years)** | |  |  |  |  |  |  |
|  | 18-19 | 91.6 | 164 (2.3) | 88.2 | 89 (2.4) | 95.7 | 75 (2.1) |
|  | 20-29 | 94.2 | 690 (9.5) | 92.9 | 366 (9.8) | 95.7 | 324 (9.1) |
|  | 30-39 | 93.6 | 1,008 (13.9) | 92.2 | 504 (13.5) | 95.0 | 504 (14.2) |
|  | 40-49 | 92.7 | 1,060 (14.6) | 92.5 | 553 (14.8) | 92.8 | 507 (14.3) |
|  | 50-59 | 90.4 | 1,305 (17.9) | 89.2 | 688 (18.4) | 91.8 | 617 (17.4) |
|  | 60-69 | 90.7 | 1,423 (19.6) | 88.9 | 712 (19.1) | 92.5 | 711 (20.1) |
|  | 70-79 | 86.1 | 1,161 (16.0) | 82.5 | 558 (15.0) | 89.4 | 603 (17.0) |
|  | 80+ | 57.8 | 462 (6.4) | 46.5 | 262 (7.0) | 72.7 | 200 (5.6) |
| **Migration background country^** | |  |  |  |  |  |  |
|  | Caribbean Netherlands | 82.6 | 58 (0.8) | 79.7 | 29 (0.8) | 85.5 | 29 (0.8) |
|  | Other Europa | 88.5 | 532 (7.3) | 86.2 | 294 (7.9) | 91.3 | 238 (6.7) |
|  | Indonesia | 82.3 | 212 (2.9) | 83.0 | 113 (3.0) | 81.5 | 99 (2.8) |
|  | Marocco | 76.6 | 77 (1.1) | 71.1 | 37 (1.0) | 81.8 | 40 (1.1) |
|  | The Netherlands | 89.8 | 5,786 (79.6) | 87.3 | 2,947 (79.0) | 92.3 | 2,839 (80.2) |
|  | Other Africa | 89.9 | 82 (1.1) | 87.9 | 43 (1.2) | 92.1 | 39 (1.1) |
|  | Other America and Oceania | 85.1 | 84 (1.2) | 82.7 | 44 (1.2) | 87.8 | 40 (1.1) |
|  | Other Asia | 89.0 | 191 (2.6) | 83.8 | 99 (2.7) | 94.5 | 92 (2.6) |
|  | Suriname | 77.7 | 123 (1.7) | 73.0 | 76 (2.0) | 85.3 | 47 (1.3) |
|  | Türkiye | 82.6 | 128 (1.8) | 83.2 | 50 (1.3) | 82.2 | 78 (2.2) |
| **Education level*** | |  |  |  |  |  |  |
|  | Primary and pre-vocational education | 79.9 | 1,879 (26.3) | 75.0 | 1,058 (28.8) | 86.3 | 821 (23.6) |
|  | Vocational and secondary education | 91.2 | 2,749 (38.5) | 90.1 | 1,331 (36.3) | 92.2 | 1,418 (40.7) |
|  | Tertiary and academic education | 93.6 | 2,521 (35.3) | 93.0 | 1,279 (34.9) | 94.2 | 1,242 (35.7) |
| **Standardized yearly household income**± | |  |  |  |  |  |  |
|  | First quintile (≤ €19.800) | 77.4 | 793 (11.1) | 73.7 | 439 (11.9) | 81.9 | 354 (10.2) |
|  | Second quintile (€19.801-€26.400) | 80.6 | 1,245 (17.4) | 76.9 | 709 (19.3) | 85.5 | 536 (15.4) |
|  | Third quintile (€26.401-€33.700) | 89.9 | 1,494 (20.8) | 87.5 | 755 (20.5) | 92.4 | 739 (21.2) |
|  | Fourth quintile (€33.701-€43.200) | 92.3 | 1,731 (24.2) | 91.6 | 858 (23.3) | 93.1 | 873 (25.1) |
|  | Fifth quintile (> €43.200) | 94.8 | 1,904 (26.6) | 93.8 | 921 (25.0) | 95.7 | 983 (28.2) |

* Until age 25 highest followed, above 25 highest achievements

^ combination of country of birth individual and parents

± based on the standardized yearly household income 2021

*Abbreviations:* n, number

**Supplement Table 7.** Mean scores of the Life’s Essential 8 metric sleep for the Dutch adult population among sociodemographic characteristics

|  | | **Total** | | **Women** | | **Men** | |
| --- | --- | --- | --- | --- | --- | --- | --- |
|  | | **Mean CVH** | **n, %** | **Mean CVH** | **n, %** | **Mean CVH** | **n, %** |
| **Sample** | |  |  |  |  |  |  |
| **Age (in years)** | |  |  |  |  |  |  |
|  | 18-19 | 76.2 | 235 (2.9) | 72.5 | 125 (3.0) | 80.5 | 110 (2.8) |
|  | 20-29 | 74.6 | 994 (12.4) | 73.8 | 539 (13.0) | 75.6 | 455 (11.8) |
|  | 30-39 | 76.5 | 1,143 (14.2) | 74.2 | 577 (13.9) | 78.9 | 566 (14.6) |
|  | 40-49 | 77.2 | 1,161 (14.5) | 74.5 | 610 (14.7) | 80.1 | 551 (14.3) |
|  | 50-59 | 72.9 | 1,387 (17.3) | 69.1 | 740 (17.8) | 77.2 | 647 (16.7) |
|  | 60-69 | 75.3 | 1,461 (18.2) | 70.9 | 740 (17.8) | 79.8 | 721 (18.7) |
|  | 70-79 | 78.2 | 1,182 (14.7) | 73.0 | 567 (13.6) | 82.9 | 615 (15.9) |
|  | 80+ | 77.2 | 463 (5.8) | 72.8 | 263 (6.3) | 83.0 | 200 (5.2) |
| **Migration background country^** | |  |  |  |  |  |  |
|  | Caribbean Netherlands | 70.8 | 62 (0.8) | 71.6 | 32 (0.8) | 70.0 | 30 (0.8) |
|  | Other Europa | 73.6 | 591 (7.4) | 69.2 | 333 (8.0) | 79.2 | 258 (6.7) |
|  | Indonesia | 74.5 | 234 (2.9) | 70.2 | 124 (3.0) | 79.5 | 110 (2.8) |
|  | Marocco | 62.7 | 89 (1.1) | 56.5 | 43 (1.0) | 68.5 | 46 (1.2) |
|  | The Netherlands | 76.7 | 6,348 (79.1) | 73.3 | 3,268 (78.5) | 80.3 | 3,080 (79.7) |
|  | Other Africa | 77.9 | 95 (1.2) | 74.3 | 49 (1.2) | 81.7 | 46 (1.2) |
|  | Other America and Oceania | 68.3 | 103 (1.3) | 70.2 | 53 (1.3) | 66.2 | 50 (1.3) |
|  | Other Asia | 71.3 | 229 (2.9) | 69.3 | 120 (2.9) | 73.5 | 109 (2.8) |
|  | Suriname | 72.8 | 137 (1.7) | 70.8 | 85 (2.0) | 76.0 | 52 (1.3) |
|  | Türkiye | 72.0 | 138 (1.7) | 68.5 | 54 (1.3) | 74.3 | 84 (2.2) |
| **Education level*** | |  |  |  |  |  |  |
|  | Primary and pre-vocational education | 73.2 | 1,911 (24.2) | 69.4 | 1,080 (26.4) | 78.1 | 831 (21.9) |
|  | Vocational and secondary education | 75.8 | 2,903 (36.8) | 71.8 | 1,399 (34.2) | 79.6 | 1,504 (39.6) |
|  | Tertiary and academic education | 77.4 | 3,082 (39.0) | 75.0 | 1,617 (39.5) | 80.1 | 1,465 (38.6) |
| **Standardized yearly household income**± | |  |  |  |  |  |  |
|  | First quintile (≤ €19.800) | 69.9 | 915 (11.6) | 68.4 | 509 (12.4) | 71.7 | 406 (10.7) |
|  | Second quintile (€19.801-€26.400) | 72.8 | 1,324 (16.8) | 69.0 | 762 (18.6) | 77.8 | 562 (14.8) |
|  | Third quintile (€26.401-€33.700) | 77.3 | 1,623 (20.5) | 73.6 | 815 (19.9) | 81.0 | 808 (21.3) |
|  | Fourth quintile (€33.701-€43.200) | 77.5 | 1,907 (24.1) | 74.5 | 955 (23.3) | 80.5 | 952 (25.0) |
|  | Fifth quintile (> €43.200) | 77.7 | 2,129 (27.0) | 74.1 | 1,056 (25.8) | 81.1 | 1,073 (28.2) |

* Until age 25 highest followed, above 25 highest achievements

^ combination of country of birth individual and parents

± based on the standardized yearly household income 2021

*Abbreviations:* n, number

**Supplement Table 8.** Distribution of the health behaviour CVH score categories across sociodemographic characteristics for the Dutch adult population

|  | | **Poor CVH**  **LE8 score 0-49** | **Intermediate CVH**  **LE8 score 50-79** | **Ideal CVH**  **LE8 score 80-100** |
| --- | --- | --- | --- | --- |
| **Sample, n(%)** | | 715 (10.3) | 3958 (56.8) | 2294 (32.9) |
| **Biological Sex, n(%)** | |  |  |  |
| Men | | 326 (9.7) | 1877 (56.0) | 1146 (34.2) |
| Women | | 389 (10.8) | 2081 (57.5) | 1148 (31.7) |
| **Age (in years), n(%)** | |  |  |  |
|  | 18-19 | 20 (12.3) | 98 (60.5) | 44 (27.2) |
|  | 20-29 | 96 (14.2) | 401 (59.3) | 179 (26.5) |
|  | 30-39 | 88 (8.9) | 570 (57.9) | 326 (33.1) |
|  | 40-49 | 95 (9.3) | 569 (55.7) | 358 (35.0) |
|  | 50-59 | 148 (11.8) | 717 (57.4) | 385 (30.8) |
|  | 60-69 | 108 (8.0) | 790 (58.3) | 458 (33.8) |
|  | 70-79 | 87 (8.0) | 578 (52.9) | 427 (39.1) |
|  | 80+ | 73 (17.2) | 235 (55.3) | 117 (27.5) |
| **Migration background country^, n(%)** | |  |  |  |
|  | Caribbean Netherlands | 11 (20.0) | 26 (47.3) | 18 (32.7) |
|  | Other Europa | 60 (11.7) | 296 (57.8) | 156 (30.5) |
|  | Indonesia | 30 (14.6) | 117 (57.1) | 58 (28.3) |
|  | The Netherlands | 509 (9.2) | 3168 (57.2) | 1863 (33.6) |
|  | Africa | 19 (12.6) | 73 (48.3) | 59 (39.1) |
|  | Other America and Oceania | 12 (15.2) | 45 (57.0) | 22 (27.8) |
|  | Other Asia | 27 (14.9) | 98 (54.1) | 56 (30.9) |
|  | Suriname | 18 (15.3) | 65 (55.1) | 35 (29.7) |
|  | Türkiye | 29 (23.0) | 70 (55.6) | 27 (21.4) |
| **Education level*, n(%)** | |  |  |  |
|  | Primary and pre-vocational education | 295 (16.5) | 1081 (60.4) | 413 (23.1) |
|  | Vocational and secondary education | 270 (10.2) | 1566 (59.2) | 810 (30.6) |
|  | Tertiary and academic education | 138 (5.7) | 1242 (51.4) | 1034 (42.8) |
| **Standardized yearly household income**±**, n(%)** | |  |  |  |
|  | First quintile (≤ €19.800) | 173 (22.9) | 421 (55.8) | 161 (26.6) |
|  | Second quintile (€19.801-€26.400) | 192 (16.0) | 698 (58.1) | 311 (30.3) |
|  | Third quintile (€26.401-€33.700) | 136 (9.5) | 828 (57.5) | 475 (36.2) |
|  | Fourth quintile (€33.701-€43.200) | 105 (6.3) | 968 (58.5) | 582 (37.4) |
|  | Fifth quintile (> €43.200) | 98 (5.4) | 984 (54.1) | 737 (42.7) |

* Until age 25 highest followed, above 25 highest achievements

^ combination of country of birth individual and parents

± based on the standardized yearly household income 2021

*Abbreviations: CVH, cardiovascular health;* n, number
